# Heteromers of amyloid precursor protein in cerebrospinal fluid

**DOI:** 10.1186/1750-1326-10-2

**Published:** 2015-01-08

**Authors:** Inmaculada Cuchillo-Ibañez, Inmaculada Lopez-Font, Alba Boix-Amorós, Gunnar Brinkmalm, Kaj Blennow, Jose-Luis Molinuevo, Javier Sáez-Valero

**Affiliations:** Instituto de Neurociencias de Alicante, Universidad Miguel Hernández-CSIC, Av. Ramón y Cajal s/n, Sant Joan d’Alacant, Spain; Centro de Investigación Biomédica en Red sobre Enfermedades Neurodegenerativas (CIBERNED), Sant Joan d’Alacant, Spain; Clinical Neurochemistry Lab, Institute of Neuroscience and Physiology, University of Gothenburg, Mölndal Campus, Gothenburg, Sweden; Alzheimer’s Disease and Other Cognitive Disorders Unit, Neurology Service, Hospital Clinic, Barcelona, Spain

**Keywords:** sAPPα, sAPPβ, Heteromers, Cerebrospinal fluid, Alzheimer’s disease, ELISA

## Abstract

**Background:**

Soluble fragments of the amyloid precursor protein (APP) generated by α- and β-secretases, sAPPα and sAPPβ, have been postulated as promising new cerebrospinal fluid (CSF) biomarkers for the clinical diagnosis of Alzheimer’s disease (AD). However, the capacity of these soluble proteins to assemble has not been explored and could be relevant. Our aim is to characterize possible sAPP oligomers that could contribute to the quantification of sAPPα and sAPPβ in CSF by ELISA, as well as to characterize the possible presence of soluble full-length APP (sAPPf).

**Results:**

We employed co-immunoprecipitation, native polyacrylamide gel electrophoresis and ultracentrifugation in sucrose density gradients to characterize sAPP oligomers in CSF. We have characterized the presence of sAPPf in CSF from NDC and AD subjects and demonstrated that all forms, including sAPPα and sAPPβ, are capable of assembling into heteromers, which differ from brain APP membrane-dimers. We measured sAPPf, sAPPα and sAPPβ by ELISA in CSF samples from AD (n = 13) and non-disease subjects (NDC, n = 13) before and after immunoprecipitation with antibodies against the C-terminal APP or against sAPPβ. We demonstrated that these sAPP heteromers participate in the quantification of sAPPα and sAPPβ by ELISA. Immunoprecipitation with a C-terminal antibody to remove sAPPf reduced by ~30% the determinations of sAPPα and sAPPβ by ELISA, whereas immunoprecipitation with an APPβ antibody reduced by ~80% the determination of sAPPf and sAPPα.

**Conclusions:**

The presence of sAPPf and sAPP heteromers should be taken into consideration when exploring the levels of sAPPα and sAPPβ as potential CSF biomarkers.

**Electronic supplementary material:**

The online version of this article (doi:10.1186/1750-1326-10-2) contains supplementary material, which is available to authorized users.

## Background

**Figure 1:**
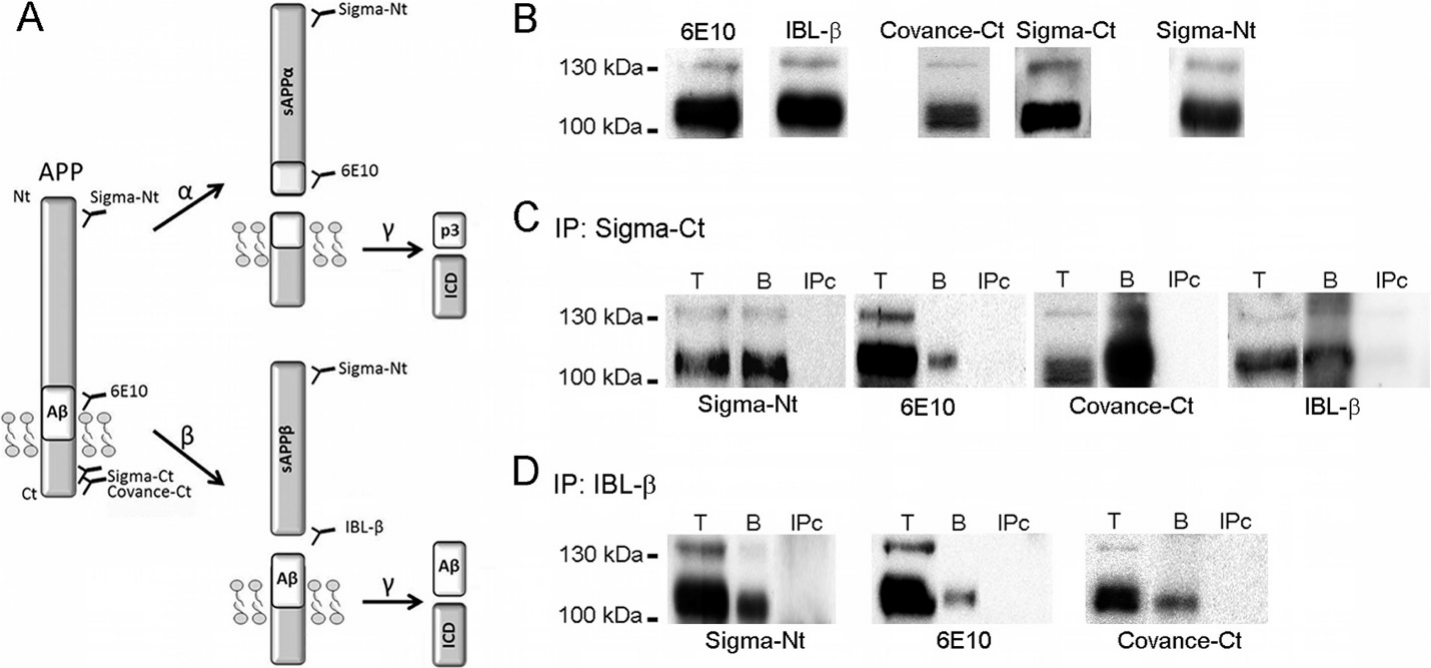
**Soluble full-length APP (sAPPf) is present in human CSF. (A)** Schematic representation of full-length APP processing by α/γ-secretase (non-amyloidogenic pathway) and β/γ-secretase (amyloidogenic pathway) and the generation of extracellular large sAPPα, sAPPβ fragments, and the shorter Aβ and p3 peptides; as well as fragments containing the C-terminal APP (CTF and ICD) (not drawn in scale). The epitopes for the anti-APP antibodies used in this study are indicated. **(B)** Western blotting of human CSF samples from non-demented controls (NDC) subjects, resolved with the indicated anti-APP antibodies. **(C, D)** CSF aliquots (Total, T) were immunoprecipitated with either the anti-C-terminal APP antibody from Sigma (**C**, Sigma-Ct) or the pan-specific IBL-β antibody (D, IBL-β). Precipitated proteins (bound fraction, B) were immunoblotted with the antibodies as indicated. In the absence of capture-antibody (IPc), no bands were observed.

Abnormal accumulation of β-amyloid peptide (Aβ) in the brain is characteristic of Alzheimer’s disease (AD). The Aβ peptide is generated by processing a large type I transmembrane spanning glycoprotein, the amyloid precursor protein (APP), through the successive action of proteolytic enzymes called secretases. Sequential processing of APP begins with either the action of α-secretase, or alternatively by β-secretase, followed by γ-secretase cleavage ([1]; see also Figure 1A). When APP molecules are cleaved by α-secretase within the Aβ domain, the generation of the Aβ peptide is precluded. When cleavage is carried out by β and γ-secretase, a 17-43 amino acid Aβ peptide is generated [2]. The Aβ40 peptide is the most abundant species, while the Aβ42 variant is the most amyloidogenic form of the peptide, associated with AD pathogenesis [3]. Cleavage by α- and β-secretase also generates soluble forms of APP, named sAPPα and sAPPβ, respectively, which are present in human CSF [4, 5] and have been postulated as potential new AD biomarkers. However, to date, no consistent change in CSF sAPPα and sAPPβ levels have been identified as associated to the AD condition [6]. Moreover, an unexpected positive correlation has been consistently reported between both forms, indicating a similar shift for sAPPα and sAPPβ levels [7–10].

Interestingly, it has been demonstrated that full-length APP containing an intact cytoplasmic domain also exists as a soluble form (sAPPf) [11, 12]. The possible contribution of sAPPf when estimating levels of large sAPP fragments in CSF has thus far not been considered, nor has the existence of APP heteromers [13] in CSF. The possibility that sAPP exists in CSF forming oligomers is particularly relevant, since most quantifications of sAPPα and sAPPβ in CSF from Alzheimer’s disease subjects rely on ELISA determinations.

In this study, we investigate the presence of sAPPf in AD CSF, as well as the oligomerization of sAPP species, to determine how this may affect the measurement of sAPPα/sAPPβ levels by currently available ELISA immunoassays.

## Results

### sAPPf is present in CSF and forms heteromers

To determine the presence of sAPPf in human CSF, we first examined samples by Western blotting using different anti-APP antibodies (Figure 1A shows a schematic representation of full-length APP, sAPP fragments generated after secretases processing and the epitopes recognized by different antibodies). The 6E10 antibody, which recognizes an epitope present in sAPPα, and absent in sAPPβ, revealed bands of approximately ~110 and 130 kDa (Figure 1B). A similar banding pattern was obtained for sAPPβ using a pan-specific antibody for the C-terminus of sAPPβ (IBL; Figure 1B). It is not possible to distinguish between sAPPα and sAPPβ by Western blotting due to their small size difference. Immunoblotting with a C-terminal APP antibody (Covance-Ct) displayed similar sAPP banding patterns, revealing a broad band of ~110 kDa, and a faint band of 130 kDa only evident in some samples (Figure 1B). An antibody against the N-terminal (Sigma-Nt) also detected bands similar in size to the immunoreactive bands detected with the previous antibodies. The specificity of these antibodies was tested and confirmed (see Additional file 1: Figure S1).

To further characterize sAPPf in human CSF, we performed immunoprecipitation/Western blotting assays (Figure 1C). CSF samples were immunoprecipitated using a C-terminal APP antibody (Sigma-Ct) and detection was performed with several antibodies. The 6E10 and Sigma-Nt antibodies confirmed the specificity of the APP signal in immunoprecipitates. The alternative C-terminal APP antibody Covance-Ct confirmed the presence of sAPPf in CSF samples (Figure 1C). The sAPPβ antibody specific for its C-terminus (IBL-β) also recognized APP forms immunoprecipitated by the C-terminal APP antibody, suggesting the presence of heteromers of sAPPf and sAPPβ containing the C-terminal. To corroborate the presence of these heteromers, CSF samples were immunoprecipitated with IBL-β, and then immunoblotted with Sigma-Nt, 6E10 and Covance-Ct antibodies (Figure 1D). Immunoblots with 6E10 and Covance-Ct demonstrated the presence of heteromers containing sAPPβ and sAPPf. Due to 6E10 also recognizes sAPPα, we do not discard the presence of heteromers containing sAPPβ and sAPPα. We do not exclude that some of the 6E10 immunoreactivity detected in Sigma-Ct immunoprecipitates arises from sAPPα forms, reflecting the presence of heteromers containing sAPPf and sAPPα. In the absence of capture-antibody (IPc), or when a rabbit IgG was used for immunoprecipitation (not shown), no bands were observed on the immunoblots (Figure 1C, D).

**Figure 2:**
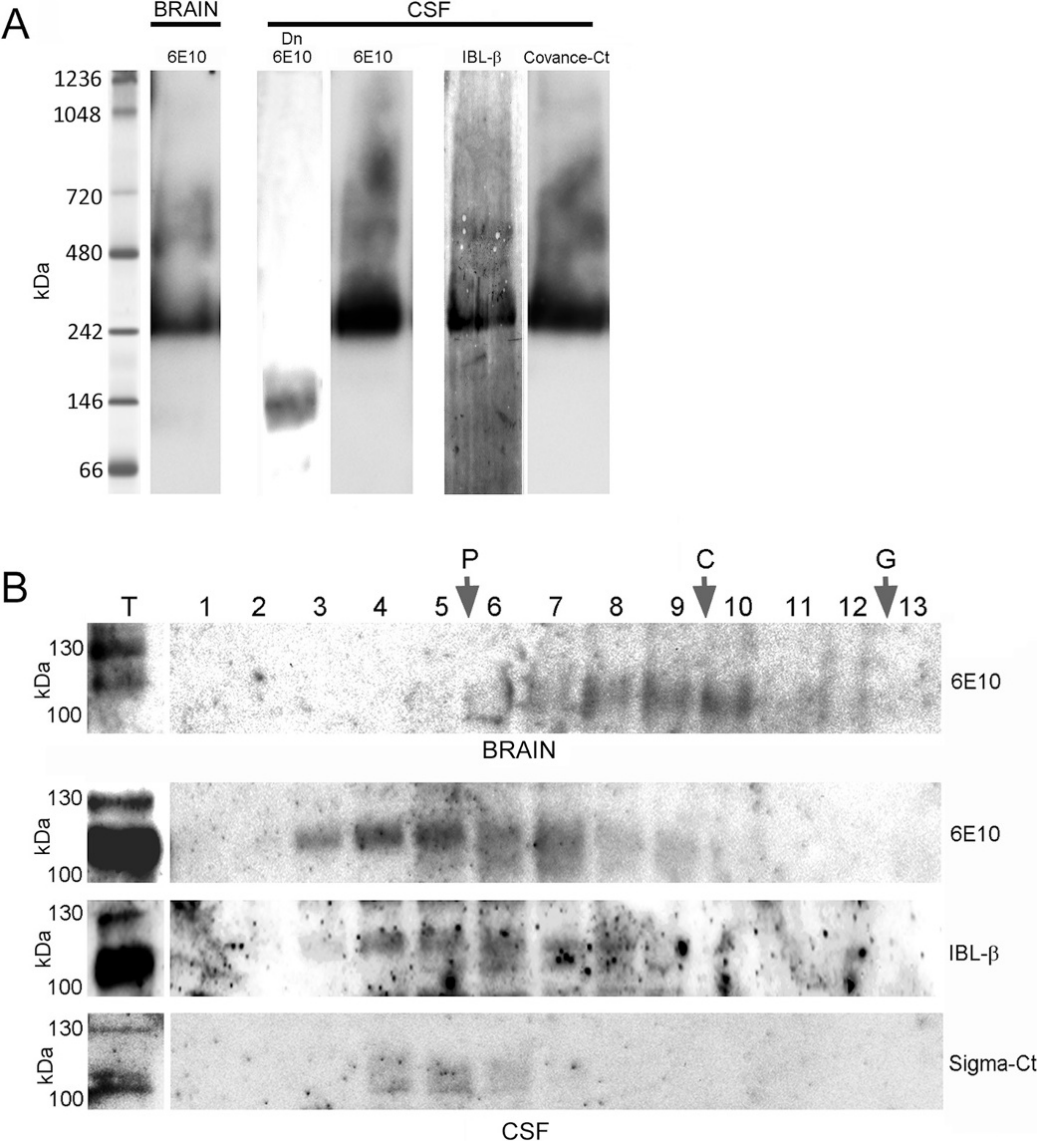
**Characterization of APP complexes by native-PAGE and sucrose gradient ultracentrifugation. (A)** APP complexes from the brain (frontal cortex from NDC subjects) and CSF samples (NDC subjects) were analyzed by blue native-PAGE. Incubation of blots with antibodies for the different APP epitopes confirmed the presence of APP dimers in brain extracts and CSF samples (~242 kDa), but also the existence of APP complexes with higher molecular weight. A CSF sample denatured by boiling at 95°C for 5 min under fully reducing conditions (Dn) was also analyzed by blue native-PAGE to warrant the migration of the monomeric sAPP band. **(B)** Brain extracts and CSF samples were also fractionated on 5-20% sucrose density gradients. The fractions (collected from the top of each tube) were immunoblotted for APP with the 6E10 antibody, and additionally with IBL-β and Sigma-Ct antibodies for CSF samples. Enzymes of known sedimentation coefficient, β-galactosidase (G, 16.0S; ~540 kDa), catalase (C, 11.4S; ~232 kDa) and alkaline phosphatase (P, 6.1S; ~140-160 kDa) were used as internal markers.

Next, we characterized sAPP oligomers from brain membranes and CSF samples by blue native-PAGE (Figure 2A). Brain samples were solubilized in buffer containing 0.5% dodecylmaltoside [14]. The major APP band in the brain was identified at ~240 kDa with the 6E10 antibody. This molecular mass corresponds to the predictable size of APP dimers. Weaker bands with high molecular mass were also identified at ~480 and 720 kDa, probably corresponding to large complexes formed by association of APP dimers. In CSF, the major band is at ~240 kDa, with smear-like bands of higher molecular mass also visible (Figure 2A), confirming that sAPP also associates in complexes. The interpretation of results from blue native-PAGE analysis of membrane protein complexes is complicated by the limitations in comparing the determinations of molecular size with the motilities of hydrophilic molecular weight markers. To resolve this, we have included the resolution of a CSF control sample denatured by boiling at 95°C for 5 min under fully reducing conditions and analyzed by blue native-PAGE. This denatured CSF sample served to locate the monomeric sAPP band and confirmed that APP-immunoreactive bands resolved under native conditions corresponded to dimers and high molecular mass complexes. Similar APP-immunoreactive banding patterns were identified in CSF with 6E10, IBL-β and Covance-Ct antibodies.

Previous studies have employed gradient centrifugation to characterize APP complexes [12]. Sucrose density gradients containing Brij 97 detergent were used to characterize APP complexes from brain membrane fractions and CSF samples (Figure 2B). The majority of APP solubilized from the human brain in the presence of dodecylmaltoside accumulated between catalase (C, sedimentation coefficient 11.4 S, molecular weight ~232 kDa) and alkaline phosphatase markers (P, sedimentation coefficient 6.1 S, molecular weight ~140-160 kDa), indicating the predominant presence of APP dimers. However, sAPP complexes in CSF appeared mainly close to alkaline phosphatase, indicating the instability of the CSF complexes resolved in sucrose gradients containing salt and detergent. Altogether, our data suggest that different APP complexes are present in CSF compared to those in the brain.

### sAPP heteromers participate in the determination of sAPPα and sAPPβ by ELISA

**Figure 3:**
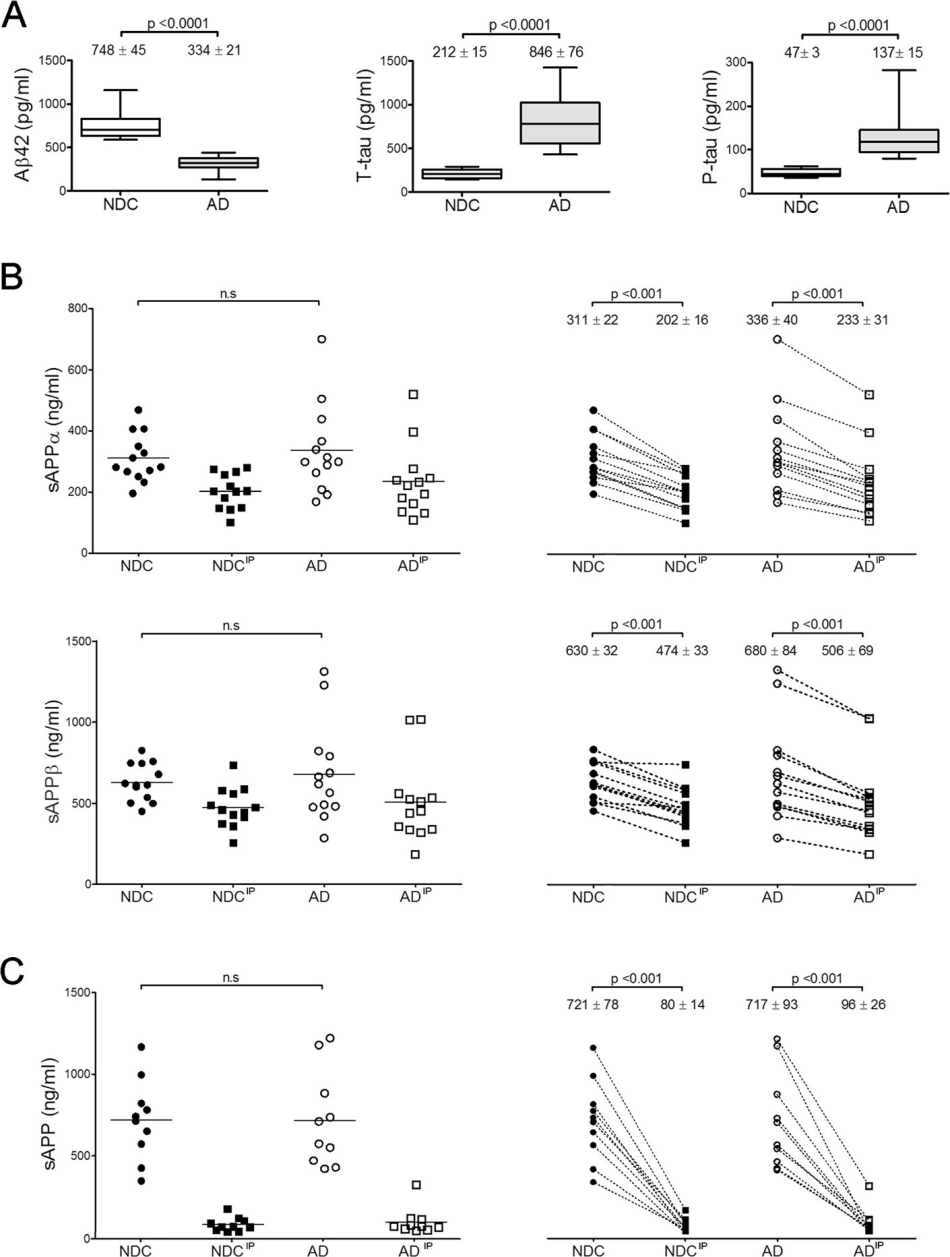
**sAPP heteromers contribute to the estimation of sAPPα and sAPPβ levels by ELISA. (A)** Box plot of CSF levels of classical AD biomarkers Aβ42, T-tau and P-tau from 13 NDC controls and 13 probable AD cases. **(B)** CSF samples were also assayed for specific sAPPα and sAPPβ using ELISA kits from IBL before (incubated with Sepharose A beads in the absence of an antibody) and after immunoprecipitation (IP) with the Sigma-Ct antibody. Before immunoprecipitation, no differences were found between NDC and AD subjects. After immunoprecipitation, levels of sAPPα and sAPPβ reduced significantly in both groups (paired Student-*t* test). **(C)** 10 fresh aliquots (from the 13 cases available) from NDC and AD groups were assayed with an alternative ELISA kit from Novex, of which the detection antibody binds to an epitope present in sAPPα and absent in sAPPβ, thus recognizing APPα and sAPPf. Before immunoprecipitation, no differences were found between NDC and AD subjects. Immunoprecipitation significantly reduced the levels of sAPPα and sAPPf in NDC and AD subjects (paired Student-*t* test). The data represent the means ± SEM (n.s., not significant).

The presence of sAPP heteromers in CSF is expected to affect the determination of sAPPα and sAPPβ by specific ELISAs. To check this possibility, we analyzed CSF samples immunoprecipitated with different antibodies to pull-down potential heteromers. CSF samples from 13 probable AD subjects, characterized for T-tau, P-tau and Aβ42 levels determined by ELISA kits (Innogenetics, Figure 3A), and 13 non-disease controls (NDC) were evaluated. We measured levels of sAPPα and sAPPβ by two specific ELISAs (the detector antibodies recognize the C-terminal of sAPPα and sAPPβ respectively, and their specificity was checked in Additional file 2: Figure S2). Samples were assayed before and after immunoprecipitation by the Sigma-Ct antibody to remove sAPP forms containing the C-terminal domain (sAPPf), which is not present in sAPPα or sAPPβ. No differences were detected in sAPPα or sAPPβ levels in either AD or NDC subjects before immunoprecipitation (Figure 3B). Consistent with other studies, a positive correlation between sAPPα and sAPPβ was observed in all samples (Additional file 2: Figure S2, r = 0.901; *p* < 0.001). After immunoprecipitation with Sigma-Ct (Figure 3B), the levels of sAPPα and sAPPβ in AD and NDC samples decreased compared to levels before immunoprecipitation (sAPPα: ~33% decrease, *p* = 0.001; sAPPβ: ~27%, *p* = 0.001). This corroborates the existence of heteromers containing sAPPf and sAPPβ, but also of sAPPf and sAPPα.

The interference of sAPP heteromers in the measurement of sAPP levels was also tested using an ELISA kit for full-length APP, which also detects sAPPα (Novex, Life Technologies). In this ELISA kit, the detector antibody recognizes an epitope located between the β-secretase and α-secretase cleavage sites, and thus, similarly to the 6E10 antibody, detects sAPPα and sAPPf, but not sAPPβ. Samples (n = 10 in both the AD and NDC samples) were analyzed before and after immunoprecipitation with the IBL-β antibody. Levels of sAPP (sAPPf and sAPPα) decreased greatly after immunoprecipitation with IBL-β in both the AD and NDC groups (~89% decrease, *p* = 0.001; Figure 3C).

Taken into account all these results, we demonstrate that the existence of sAPP as heteromers in CSF contributes to the estimation of sAPPα and sAPPβ levels by ELISA.

### Levels of sAPPf in AD CSF

**Figure 4:**
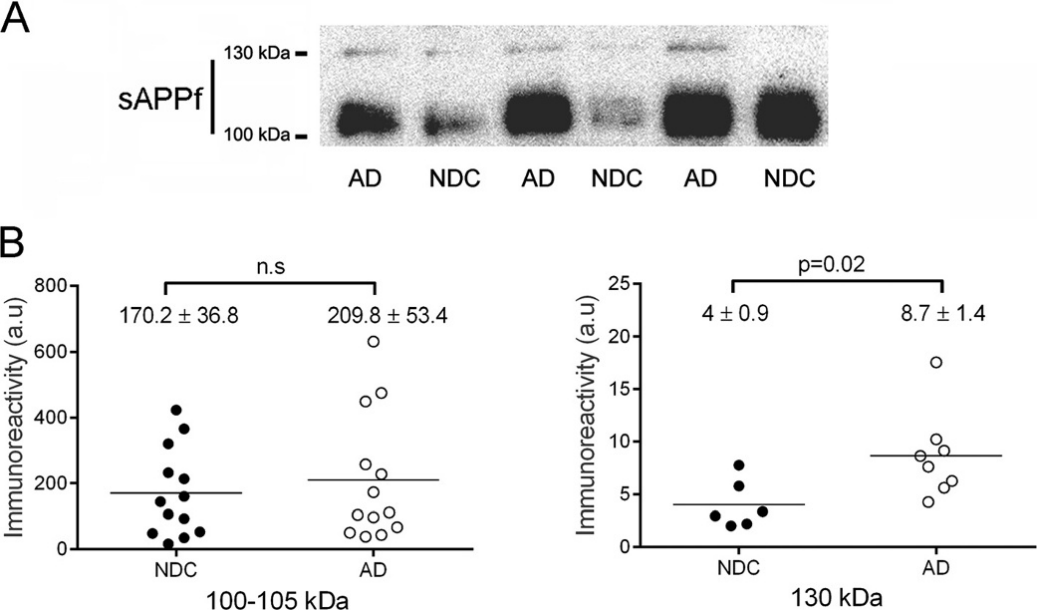
**sAPPf immunoreactivity levels in CSF from NDC and AD subjects.** Immunodetection of sAPPf in CSF samples from 13 NDC and 13 AD subjects. **(A)** Representative blot of CSF-sAPPf resolved with the Covance-Ct antibody. The 130 kDa band was not easily visualized in all samples, and was quantified at longer exposure than the 100 kDa band. **(B)** Densitometric quantification of the APP-immunoreactive bands, ~110 and 130 kDa, from the NDC and AD cases (performed in duplicate). A control CSF sample was run on different gels (processed in parallel) to normalize the immunoreactive signal between immunoblots. Differences were found only for the 130 kDa band between NDC and AD subjects. The data represent the means ± SEM (n.s., not significant).

Finally, to assess whether sAPPf levels are altered in AD, we analyzed by Western blotting fresh aliquots of the same CSF samples previously characterized for classical biomarkers (see Figure 3A). To prevent interference of sAPP heteromers, the electrophoresis was performed under denaturing conditions. sAPPf was detected with the Covance-Ct antibody (Figure 4A). Immunoreactivity of the major broad band, ~110 kDa, was not significantly different in AD compared to NDC subjects (Figure 4B). The faint 130 kDa band was not detected in all samples. In some cases where the quantification was possible, the intensity of the 130 kDa band was quantified and found to be increased by ~115% in AD (n = 8) compared to NDC subjects (n = 6; *p* = 0.02). Interestingly, the levels of sAPPf correlated with Aβ42 in the AD group (r = 0.739; *p* = 0.004), but not in the NDC cases. There was no association of sAPPf levels with age or gender.

## Discussion

In this report, we confirm the presence of sAPPf in human CSF and characterize that sAPP species associate in heteromeric complexes. Our studies demonstrate that sAPP heteromers contribute to the estimation of sAPPα and sAPPβ levels by current standard ELISA methods.

Membrane-associated APP is usually detected as a group of 110-130 kDa proteins, and sAPPf in human CSF is expected to have an apparent molecular mass similar to that of cellular APP [11], while sAPPα or sAPPβ fragments are predicted to be ~5-10 kDa smaller than full-length APP [5]. These small size differences make it difficult to distinguish sAPPf from sAPPα or sAPPβ (see Additional file 1: Figure S1). Moreover, there are three APP isoforms expressed in the brain resulting from alternative exon splicing [15]; APP695, the predominant isoform in neurons consisting of 695 amino acids, and APP751 and APP770, both isoforms harboring an amino acid insert homologous to a Kunitz-type trypsin inhibitor (KPI) [16]. Therefore, the expected sAPP banding pattern in CSF is very complex, with three potential variants of each sAPPα and sAPPβ originated from APP695, 751 and 770 isoforms. Our present study adds complexity to this scenario, demonstrating that sAPPf is also present in human CSF.

In our study, no statistical differences were observed in the level of the broad sAPPf band of 110 kDa (attributable by its molecular mass to APP695) between probable AD patients and non-demented controls; however, its levels correlated with Aβ42 levels in the AD group; this may indicate an association with the pathological levels of Aβ42, and requires further research. The 130 kDa band (which should correspond to APP751/770) was found to be increased in AD CSF. Despite the low number of samples analyzed, and the difficulty in quantifying the immunoreactivity of the 130 kDa band, our data suggest that the analysis of KPI-containing sAPPf forms may be of particular interest and needs to be more specifically addressed. Interestingly, the proportion of KPI-containing isoforms appears significantly elevated in the soluble subcellular fraction of AD brains [17].

The origin or functional significance of sAPPf in CSF is currently not known. Early studies failed to demonstrate the presence of sAPP isoforms containing its C-terminal domain in human CSF [4], but later work suggested that sAPPf exists in this fluid [11], in plasma [18] and can be released from platelets [18, 19], chromaffin cells [11, 12] and different cell lines [11, 20]. In chromaffin cells, it has also been proposed that sAPPf could be released from a non-transmembrane APP population [12]. In cell culture, the levels of sAPPf are modulated by cell depolarization in a Ca^2+^-dependent manner, as well as by cholinergic receptor agonists and antagonists [11]. A mechanism for sAPPf to appear in CSF has yet to be determined, but passive release from brain cells or neuronal cell death may be possible, as recently observed for BACE1 [21]. Full-length BACE1 and other membrane resident protein, such as presenilin-1, are present in CSF [22].

Previous studies indicate that cellular APP may form high molecular-weight APP complexes [13, 23]. Co-immunoprecipitation and native gel analysis indicated an interaction among all sAPP forms in CSF, and their assembly into APP heteromeric complexes. This could be due to the self-association properties of APP by hydrophobic and/or ionic interactions resulting in the formation of heteromeric sAPP complexes which contain several forms of sAPP, including sAPPf. Thus, CSF heteromers differ from brain APP membrane-homodimers. Native gel experiments suggest the existence of large sAPP heteromers in CSF. Interestingly, the sAPPf forms released from platelets are more susceptible to sedimentation by ultracentrifugation than sAPP forms lacking the carboxyl terminal [18]; this could indicate a different degree of oligomerization or differences in the conformation of soluble aggregates compared to cellular dimers. Here, we observed differences during ultracentrifugation in sucrose density gradients containing Brij 97 detergent, where APP complexes from the CSF appear to be more unstable than brain APP dimers.

The nature and composition of sAPPf oligomers in CSF, as well the possibility that other proteins could also be part of the sAPP complexes, have yet to be deciphered. Based on our co-immunoprecipitation and ELISA analysis, we assume that all the possible sAPP heteromers are present in CSF.

Previous reports on the levels of sAPP in CSF draw a complex scenario. A strong positive correlation between sAPPα and sAPPβ levels has been consistently found in healthy elderly individuals and AD subjects in our and other studies [6, 7, 9, 10, 24]. Since the production of sAPPβ should be inversely proportional to that of sAPPα, this is an unpredicted finding. The changes in APP gene expression levels in the brain of AD subjects are modest [25, 26], and do not to explain the concurrent positive correlation in sAPPα and sAPPβ levels. It has been suggested that there may be a common factor that regulates the metabolism of APP by influencing the activity of both α- and β-secretases or by a mutual influence on both secretases [6]. Cross-reactivity between sAPPα and sAPPβ assays has also been suggested as a contributing factor for their unexplained correlation [27]; however, development of assays based on pan-specific antibodies further confirms this positive correlation. Instead, we cannot totally exclude the possibility that cross-reactivity also contribute in our determination of sAPP heteromers; although our results indicate that such a strong correlation may be explained by the presence of heteromers containing sAPPβ and sAPPα. These sAPPβ/sAPPα heteromers would contribute proportionally to the determinations of both sAPPα and sAPPβ by ELISAs, leading to this positive correlation.

In the same line, studies of sAPPα and sAPPβ levels in CSF likely would reflect potential changes in the balance between non-amyloidogenic and amyloidogenic pathways, but reports are also contradictory. Studies of sAPPα levels in CSF from AD subjects range from an increase [8, 27–29], to no significant change [30–33], or a slight decrease [4, 34–37], compared to levels in NDC subjects. Similarly, some reports indicate that sAPPβ levels increase in AD compared to NDC cases [27], but others show no change or even a decrease [7, 32, 35, 37]. Simultaneous determination in CSF of sAPPα levels with respect to sAPPβ could potentially reflect APP brain metabolism, but comparative studies have again resulted controversial. Given that the α-secretase pathway is the predominant APP processing pathway, higher levels of sAPPα would be expected compared to sAPPβ levels in CSF from non-disease individuals. Some studies have reported higher levels of CSF sAPPα than sAPPβ in non-disease controls [11, 32], although most published reports do not support these results [7, 10, 24, 27]. Our results demonstrate that the presence of sAPPf, detectable by some ELISAs developed for sAPPα, should be considered a contributing factor for these contradictory findings. Thus, comparison of sAPPβ and sAPPα levels by IBL ELISA kits (using specific anti-APP antibodies which recognize the C-terminal of sAPPα or sAPPβ) showed higher amounts of sAPPβ in CSF than that of sAPPα. Nonetheless, comparison of sAPPβ with those sAPP levels obtained with the Novex Life kit does not confirm this appreciation. The Novex Life kit, like other kits based on the 6E10 antibody [11, 32], is based on an antibody recognizing the N-terminal part of the Aβ peptide for detection, thus binding sAPPf and sAPPα. The contribution of sAPPf to the estimation of sAPPα levels will explain discrepancies between the Novex Life and IBL kits. Altogether, our data suggest that sAPPβ is more abundant in CSF than sAPPα, and that sAPPf is the least represented. However, since all those ELISA kits do not discriminate between homodimers, heterodimers and large heteromers, an analysis of sAPPα and sAPPβ under conditions that favor the measurement of sAPP isoforms in their monomeric state is warranted to estimate their correct potential as AD biomarkers.

Finally, we have speculated about the abundance of sAPP dimers by our ELISA assays, taking into account that from these data we cannot discriminate sAPP dimers from large sAPP oligomers. Based on the Novex Life ELISA analysis after IBL-β immunoprecipitation, we presumed that the most abundant oligomers include sAPPβ, probably being sAPPα/sAPPβ, since the oligomers which have not been pulled-down, sAPPf/sAPPα, sAPPα/sAPPα and/or sAPPf/sAPPf, constitute altogether ~10% of the non-immunoprecipitated samples. Immunoprecipitation with the Sigma-Ct antibody followed by ELISA analysis for sAPPα and sAPPβ (IBL) revealed in both cases that sAPPf/sAPPα or sAPPf/sAPPβ heteromers represent ~30% of oligomers that include sAPPα and sAPPβ. We speculate that the sAPPα/sAPPα, sAPPf/sAPPf, but also sAPPβ/sAPPβ homodimers, would constitute the least abundant population. In any case, specific characterization of each sAPP oligomer in CSF, and potential differences in the AD condition, will require further research.

## Conclusions

The use of CSF biomarkers for AD in clinical practice is challenging due to the high variability in their levels. Such variability has been partially attributed to different pre-analytical and analytical procedures between laboratories, highlighting the need to establish standardized operating procedures [38, 39]. CSF-sAPPα and sAPPβ have been proposed as promising CSF biomarkers for AD and other neuropathological conditions [40–45], but have failed to meet expectations with their often contradictory findings. We describe that CSF-sAPP forms, including soluble full-length APP, assemble into oligomers and that heteromers contribute to the estimation of sAPP levels by ELISAs, and thus, potentially account for the underestimation of real sAPP levels. Our finding could explain the controversy about CSF-sAPPα and sAPPβ levels, and may change how these biomarkers are assessed. These findings indicate the need to design new assays for CSF-sAPP, with new conditions to selectively analyze sAPP monomers, which could unmask pathological differences, and to develop new approaches for sAPP heteromers, which could reveal new biomarkers.

## Material and methods

### Patients

This study was approved by the ethics committee of the Miguel Hernandez University and was carried out in accordance with the Declaration of Helsinki. Lumbar CSF samples were obtained from 13 patients with probable Alzheimer’s disease (AD; 5 men and 8 women, 67 ± 2 years), and 13 healthy volunteers, non-demented controls (NDC; 5 men and 8 women, 62 ± 3 years) from the Hospital Clinic (Barcelona, Spain). All AD patients fulfilled the NINCDS-ADRDA criteria for “probable” AD [46]. Control subjects had no history or symptoms of neurological or psychiatric disorders or memory complaints.

### Brain extracts

Brain frontal cortex samples (4 NDC cases, 75 ± 7 years; post-mortem interval 4-8 hours) were obtained from the UIPA neurological tissue bank (Unidad de Investigación Proyecto Alzheimer; Madrid, Spain). Samples were solubilized in 20 mM HEPES pH 7.5, 50 mM KCl, 2 mM EDTA and ultracentrifuged at 300,000 × *g* for 1 h. The pellet was homogenized in 0.5% dodecylmaltoside, 20% glycerol, and 25 mM bis-Tris pH 7.0. After 45 min in continuous mixing at 4°C followed by ultracentrifugation at 200,000 × *g* for 30 min, the supernatant was collected and frozen at -80°C.

### Cell culture

CHO cells over-expressing wild-type human APP-751 [47] were cultured in Opti-MEM supplemented with 10% FBS, G-148 (200 μg/ml) and puromycin (2.5 μg/ml).

### Western blotting

Samples of CSF (30 μL) were boiled at 95°C for 5 min and electrophoresed in 7.5% SDS-PAGE. sAPP species were detected using the following antibodies: anti-APP C-terminal (monoclonal, 1:2,000; Covance, Princeton, NJ, USA; named here Covance-Ct), anti-APP C-terminal (polyclonal, 1:2,000; Sigma Aldrich, St. Louis, MO, USA; named here Sigma-Ct), anti-APP 6E10 (which reacts with both sAPPf and sAPPα, as well as with free Aβ; monoclonal, 1:2,000; Covance; named here 6E10), anti-APP N-terminal (polyclonal, 1:2,000; Sigma Aldrich; named here Sigma-Nt), anti-sAPPβ (specific for the C-terminus of sAPPβ; polyclonal, 1:50; IBL, Hamburg, Germany; named here IBL-β), and anti-sAPPα (specific for the C-terminus of sAPPα; monoclonal, 1:50; IBL; named here IBL-α). When appropriate, a control CSF sample was used to normalize the immunoreactive signal between immunoblots. The signal was visualized by Luminata^TM^ Forte (Merck-Millipore, Feltham, UK) and analyzed using Science Lab Image Gauge v4.0 software (Fujifilm).

To test the specificity of the APP antibodies, aliquots of conditioned media from CHO cells over-expressing APP-751 were resolved by simultaneous detection of immunoreactivity to two-antibodies (using a different combination of antibodies: Covance-Ct/IBL-β, Sigma-Ct/6E10 and 6E10/IBL-β). Blots were then probed with the appropriate conjugated secondary antibodies (IRDye 800CW goat anti-mouse and IRDye 680RD goat anti-rabbit; LI-COR Biosciences GmbH, Bad Homburg, Germany) and imaged on an Odyssey Clx Infrared Imaging System (LI-COR). Band intensities were analyzed using LI-COR software (Image Studio Lite).

### Blue native gel analysis

The Life Technologies NativePAGE Novex 4-16% Bis-Tris Gel System was used in accordance with the manufacturer’s protocol. Samples were prepared in a 30 μl total volume containing 7.5 μL of NuPage LDS sample buffer (4×), 3 μl of 5% G-250 sample additive and 1.5 μl of DDM 10% (n-dodecyl-β-D-maltoside). The NativeMark™ unstained protein standard (Life Technologies, Carlsbad, CA, USA) was used as a protein ladder.

### Immunoprecipitation

Immunoprecipitations were performed at 4°C by incubating 150 μL of CSF overnight with 120 μL Sepharose A beads coupled to the specific APP antibody. Precipitated proteins were eluted with 0.1 M glycine buffer at pH 2.5. Immunoprecipitation controls were performed in Sepharose A beads, but absent of any antibody. After pH neutralization with 1 M Tris-HCl pH 9.5, the supernatants were denatured in Laemmli sample buffer at 95°C for 5 min. Preliminary assays resolved by ELISA proved the lack of non-specific binding of sAPP to Sepharose A beads (without an antibody), compared with aliquots samples assayed directly (not incubated in the presence of Sepharose A beads or any antibody).

### Sucrose gradients

CSF samples and brain extracts were analyzed for APP complexes by ultracentrifugation at 250,000 × *g* in a continuous sucrose density gradient (5-20%). CSF aliquots (150 μL) and brain extracts (150 μl) were carefully loaded onto the top of the gradient containing 2 mL of 0.15 M NaCl, 50 mM MgCl_2_ and 0.5% Brij 97, in 50 mM Tris-HCl (pH 7.4). After centrifugation for 4 hr at 4°C in a Beckman TLS 55 rotor, approximately 13 fractions were carefully collected from the top of the tubes. Enzyme markers of known sedimentation coefficient, β-galactosidase, catalase and alkaline phosphatase were used.

### Measurement of sAPPα and sAPPβ by ELISA

A commercial ELISA kit from IBL (Hamburg, Germany) was used to quantify sAPPα (#27734) and sAPPβ (#27735) in CSF. These assays are based on a solid-phase sandwich ELISA using specific anti-APP antibodies which recognize the C-terminal of sAPPα and sAPPβ. The manufacturer estimated that cross-reactivity between sAPPα and sAPPβ fragments in each kit is less than 1.5%, and that both assays do not cross-react with full-length APP. A commercial ELISA kit for sAPPf (Novex, Life-Technologies, Carlsbad, CA, USA, #KHB0051) was also employed. This assay is based on an N-terminal APP capture-antibody and an antibody recognizing the N-terminal part of the Aβ peptide for detection, thus binding sAPPf and sAPPα, but not sAPPβ.

### Measurement of T-tau, P-tau and Aβ42 by ELISA

Total tau (T-tau), phosphorylated tau (P-tau) and Aβ1-42 (Aβ42) in CSF were determined using specific ELISA (Innogenetics, Ghent, Belgium).

### Statistical analysis

All data were analyzed using SigmaStat (Version 2.0; SPSS, Inc.) by a Student’s *t* test (two-tailed) or a Mann-Whitney *U* test for single pairwise comparisons and determination of exact *p* values. Results are presented as means ± SEM. Correlation between variables was assessed by linear regression analyses. *p* values < 0.05 were considered significant.

## Electronic supplementary material

Additional file 1: Figure S1: SDS-PAGE analysis and fluorescence detection of sAPP species. For the analysis of sAPP species, and to probe the specificity of the anti-APP antibodies, aliquots of conditioned media from CHO cells over-expressing APP-751 were analyzed by SDS-PAGE and resolved with two different anti-APP antibodies simultaneously. The fluorescence of the secondary antibodies (RDye 800CW goat anti-mouse, green; IRDye 680RD goat anti-rabbit, red) was detected with the Odyssey CLx Infrared Imaging system (LI-COR). (A) sAPPf was resolved with Covance-Ct and sAPPβ was resolved with the IBL-β antibody. (B) sAPPf was resolved with Sigma-Ct and sAPPf and sAPPα were resolved with the 6E10 antibody. (C) sAPPf and APPα were detected with 6E10 and sAPPβ was resolved with IBL-β. (D) sAPPα was resolved with the IBL-α antibody and sAPPβ was resolved with the IBL-β antibody. Simultaneous fluorescence serves to demonstrate the specificity of the C-terminal, IBL-β and IBL-α antibodies. Image showing co-localization (yellow) was only evident combining the C-terminal antibody Sigma-Ct with 6E10, whose epitope, located between the β-secretase and α-secretase cleavage sites, is present in both sAPPα and sAPPf. (JPEG 320 KB)

Additional file 2: Figure S2: Positive correlation between sAPPα and sAPPβ in CSF samples. CSF samples from 13 NDC (closed circle) and 13 AD subjects (open circle) were assayed for sAPPα and sAPPβ fragments using specific ELISA kits (see Figure 3). When data from NDC and AD were analyzed together, a strong positive correlation between sAPPα and sAPPβ levels was found (regression line for all the CSF samples is represented by a solid line; r = 0.901; *p* < 0.001). This correlation remained significant when data were analyzed separately for AD subjects (regression line is represented by a dotted line; r = 0.911, *p* < 0.001), or NDC subjects (regression line is not represented; r = 0.896, *p* < 0.001). (JPEG 236 KB)

## Abbreviations

CSF: Cerebrospinal fluid
sAPP: Soluble amyloid precursor protein
sAPPα: sAPP fragment generated after α-secretase cleavage
sAPPβ: sAPP fragment generated after β-secretase cleavage
sAPPf: soluble full-length APP
P-tau: Phospho-tau
T-tau: Total tau
AD: Alzheimer’s disease
Aβ: β-amyloid peptide, ELISA, enzyme-linked immunosorbent assay.
