# Supplementary figures and images for: Heteromers of amyloid precursor protein in cerebrospinal fluid
Source: Mol Neurodegener. 2015 Jan 8;10:2. doi: 10.1186/1750-1326-10-2 (PMC4298044; doi:10.1186/1750-1326-10-2)

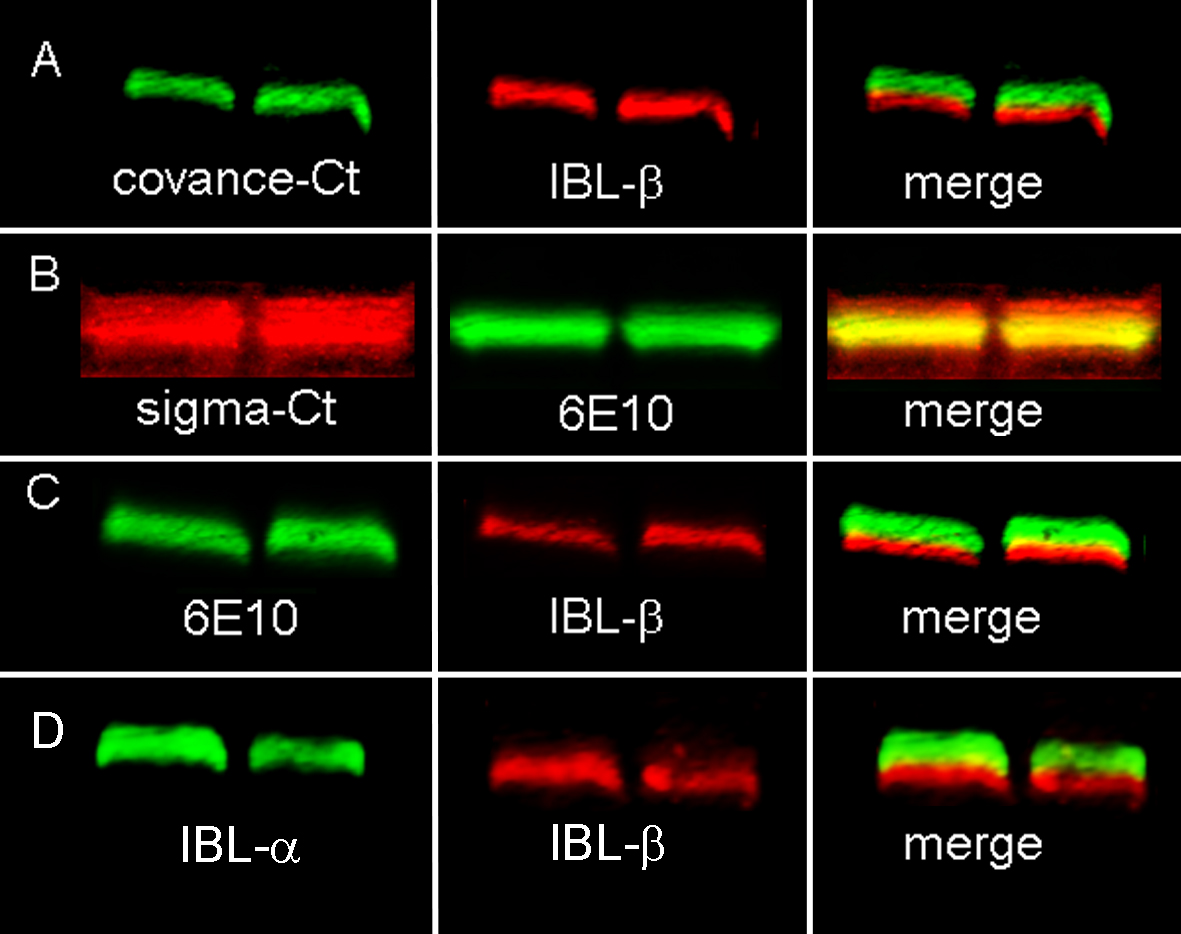

Supplement: Supplementary file 1 — Additional file 1: Figure S1: SDS-PAGE analysis and fluorescence detection of sAPP species. For the analysis of sAPP species, and to probe the specificity of the anti-APP antibodies, aliquots of conditioned media from CHO cells over-expressing APP-751 were analyzed by SDS-PAGE and resolved with two different anti-APP antibodies simultaneously. The fluorescence of the secondary antibodies (RDye 800CW goat anti-mouse, green; IRDye 680RD goat anti-rabbit, red) was detected with the Odyssey CLx Infrared Imaging system (LI-COR). (A) sAPPf was resolved with Covance-Ct and sAPPβ was resolved with the IBL-β antibody. (B) sAPPf was resolved with Sigma-Ct and sAPPf and sAPPα were resolved with the 6E10 antibody. (C) sAPPf and APPα were detected with 6E10 and sAPPβ was resolved with IBL-β. (D) sAPPα was resolved with the IBL-α antibody and sAPPβ was resolved with the IBL-β antibody. Simultaneous fluorescence serves to demonstrate the specificity of the C-terminal, IBL-β and IBL-α antibodies. Image showing co-localization (yellow) was only evident combining the C-terminal antibody Sigma-Ct with 6E10, whose epitope, located between the β-secretase and α-secretase cleavage sites, is present in both sAPPα and sAPPf. (JPEG 320 KB) [file 13024_2014_571_MOESM1_ESM.jpeg]

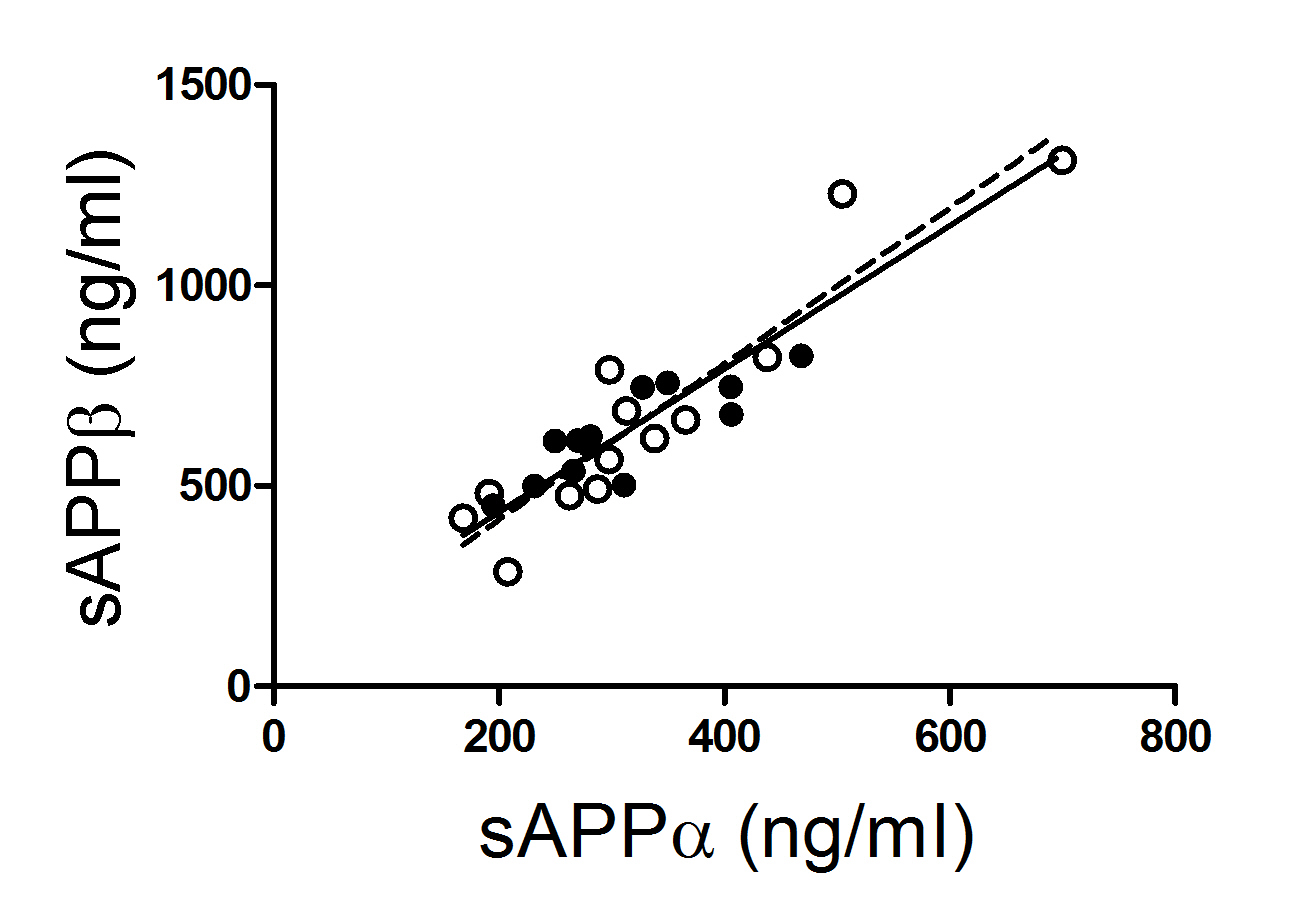

Supplement: Supplementary file 2 — Additional file 2: Figure S2: Positive correlation between sAPPα and sAPPβ in CSF samples. CSF samples from 13 NDC (closed circle) and 13 AD subjects (open circle) were assayed for sAPPα and sAPPβ fragments using specific ELISA kits (see Figure 3). When data from NDC and AD were analyzed together, a strong positive correlation between sAPPα and sAPPβ levels was found (regression line for all the CSF samples is represented by a solid line; r = 0.901; p < 0.001). This correlation remained significant when data were analyzed separately for AD subjects (regression line is represented by a dotted line; r = 0.911, p < 0.001), or NDC subjects (regression line is not represented; r = 0.896, p < 0.001). (JPEG 236 KB) [file 13024_2014_571_MOESM2_ESM.jpeg]
